# The Multiple Mechanisms of Cell Death Triggered by Resveratrol in Lymphoma and Leukemia

**DOI:** 10.3390/ijms15034977

**Published:** 2014-03-20

**Authors:** Raffaele Frazzi, Marco Tigano

**Affiliations:** Translational Research Laboratory, Department of Research and Statistics, IRCCS Arcispedale S.Maria Nuova, 42123 Reggio Emilia, Italy; E-Mail: marco.tigano@asmn.re.it

**Keywords:** resveratrol, non-Hodgkin lymphoma, apoptosis

## Abstract

Lymphoma and leukemia represent a serious threat to human health and life expectancy. Resveratrol is, among the natural-derived chemopreventive molecules, one of the most effective and better studied. In this paper the main mechanisms of cell death triggered by- or linked to- resveratrol are reviewed and discussed. The main focus is on lymphoma and leukemia experimental models where resveratrol has been tested and investigated at the cellular, molecular or physiological levels. The most relevant *in vivo* challenges involving resveratrol are also reported and analyzed in order to define the key features of this polyphenol and the potential for the treatment of hematologic tumors.

## 1. Introduction

Resveratrol (RSV) is a natural polyphenol belonging to the class of stilbenes (3,5,4′-trihydroxystilbene). It was first isolated from the roots of white hellebore in 1940 and later, in 1963, from the roots of *Polygonum Cuspidatum*, a plant already used in the Chinese and Japanese traditional medicine [1,2].

The noteworthy list of beneficial effects demonstrated on eukaryotic organisms and human beings explains the great interest raised by this compound. These positive effects include anti-oxidant and anti-aging properties, improvement of insulin sensitivity, reduction of cardiovascular disease risk and chemoprevention in cancer, among others [3]. Interestingly, RSV has also been postulated to be a mimetic of the effects of caloric restriction (CR). CR is a nutritional protocol that contemplates an average reduction of 40% in caloric intake and that has been proven to lead to the elongation of lifespan in several animal models, including rodents [4]. RSV was demonstrated to extend lifespan of yeast, worms and flies as well as CR [5,6]. The linkage between RSV and life extension through an effect on the animal metabolism is therefore plausible, even though the mechanisms involved have yet to be defined exactly at the molecular level.

The recent scientific literature reports the RSV antiproliferative and pro-apoptotic activity against a great variety of human cancer cell lines spanning from colon to prostate, from breast to lymphoma, from mesothelioma to leukemia [7–12].

Many mechanisms of action have been postulated in order to explain the antiproliferative activity of RSV. These include the activation of the intrinsic apoptotic pathway, the mitochondrial release of cytochrome c and the involvement of Bax, the generation of reactive oxygen species (ROS), the modulation of p53 pathway and the activation of the extrinsic death receptor pathway [13]. Specifically, RSV intereferes with the mitochondrial respiratory chain, and leads to the increase of ROS production [14]. The redox state of the cells plays a role in many types of apoptosis and the ROS produced at the level of the mitochondria can be involved in cell death [15]. The modulation of antioxidant enzymes can explain RSV’s ability to inhibit DNA damage in human lymphocytes induced by various toxic drugs and its ability to function as chemopreventive agent [16–18]. The current opinion is that RSV can work as a pro-oxidant as well as an anti-oxidant agent depending on the concentration administered to the cells and on the cell types [13]. For instance, the exposure of leukemia cells to sub-lethal concentrations of RSV exerts a protective action that results in the inhibition of drug-induced apoptosis [19]. The phenomenon of cytoprotection at low doses and cytotoxicity at high doses is called “hormesis”. Hormesis describes the bi-phasic dose-response that is common not only to RSV but also to many phytochemicals [20].

RSV can induce apoptosis in several types of cancer cells also through the modulation of the proteins belonging to the Bcl-2-family. RSV acts by neutralizing anti-apoptotic proteins while inducing the protein expression, conformational changes and cellular redistribution of the pro-apoptotic proteins of the Bcl-2-family [13]. This topic will be discussed further in this paper.

Indeed, RSV is a chemically well known molecule that exerts pleiotropic effects. That is to say, several molecular targets are affected by RSV treatment and the resultant pleiotropic activity explains the diverse mechanisms of action that have been described thus far.

The aim of this paper is the review of the mechanisms of cell death triggered by or linked to RSV on tumor cells of hematologic origin. We also summarize the few clinical studies concerning the use of RSV on human beings in order to provide relevant information about the actual mechanisms of cell death triggered in human tissues. These studies also underline the most critical aspects emerging from the translation to humans. The results are discussed in light of a possible future application of RSV to hematologic malignancies.

## 2. RSV in Non-Hodgkin Lymphoma and Leukemias

To date the literature describing the effects of RSV in Non-Hodgkin lymphomas (NHL) and leukemias is quite rich. The first scientific evidence demonstrating the antiproliferative and pro-apoptotic properties of RSV on these tumors were published starting from 2000 [21–24].

First, the changes on the cell cycle progression were reported. These include the accumulation in the S phase followed by the dose-dependent apoptosis onset, demonstrated by the increase of the sub-G1 peak and by the increase of Annexin^+^ (AnnV^+^) cells [21]. Accordingly to these authors, RSV induced a Fas-independent and Caspase-8 (Casp-8) independent apoptosis in the T-cell derived lymphocytic leukemia cell line CEM-C7H2. Furthermore, in the myeloid leukemia cell line HL-60, RSV can kill the cells resistant to CD95/Fas-mediated cell death, confirming that RSV acts through a Fas-independent mechanism [22]. Interestingly, the mitochondrial membrane depolarization and the Casp-9 activation were involved in the RSV-mediated cell death of several Acute Lymphoblastic Leukemia (ALL) cell lines [22].

This evidence point to a role of Caspase-9 (Casp-9) as the initiator caspase able to trigger Caspase-3 (Casp-3) cleavage following RSV treatment also in lymphoma cells. Cytochrome c is released from the mitochondria after Bax homo-oligomerization in colon and breast cancer cells treated with RSV, as recently demonstrated by Gogada and co-workers [25]. This is consistent with previous data showing that the mitochondrial membrane potential is lost as a consequence of RSV in several ALL lineages [22] and that this is the leading mechanism of the intrinsic apoptotic pathway activation.

The proteins of the Bcl-2 family are heavily affected by RSV in leukemia and lymphoma cells. WSU-CLL and ESKOL cell lines and lymphocytes from patients affected by B-cell chronic lymphocytic leukemia were killed by RSV through apoptosis while the iNOS and Bcl-2 anti-apoptotic proteins were down-regulated [26]. In the same fashion, the promyelocytic leukemia-derived cell line HL-60 was killed by apoptosis and Bcl-2 expression down-regulated by RSV [27]. In the Burkitt’s lymphoma cell line Ramos, RSV down-regulated the two anti-apoptotic proteins Bcl-X_L_ and Mcl-1 while it up-regulated the pro-apoptotic proteins Bax and Apaf-1 [28]. Notably, in the same study, the human peripheral blood mononuclear cells (PBMCs, both quiescent or mitogenically stimulated) did not show any toxicity after being treated with the same concentrations of RSV (10 μM). Also in chronic myeloid (K562) and in acute lymphoblastic (HSB-2) leukemia cells, RSV induced cell growth inhibition and apoptosis through the increase of the pro-apoptotic Bcl-2 member Bax and the cytochrome c release [29]. Along with the just described bi-functional activity on Bcl-2-family members, RSV induces conformational changes and cellular redistribution of Bax and Bak. RSV is able to trigger the exposure of the Bax *N*-terminus and its translocation to mitochondria in colon cancer cells and in leukemia cells [30,31]. The exposure of the *N*-terminus of Bax and Bak seems to be required for the translocation to mitochondria and the induction of apoptosis.

Casp-3 and STAT3 phosphorylation are also involved during RSV-mediated cell death of T-lymphocytes infected with human T-cell leukemia virus type 1 (HTLV-1) [32]. In these cell lines (MT-2 and HUT-102) RSV induces the cleavage of casp-3 and poly(ADP-ribose) polymerase indicating a caspase-dependent apoptosis. In the same system of adult T cell leukemia, myeloid cell leukemia sequence (Mcl-1) and cellular inhibitor of apoptosis protein (cIAP)-2 were inhibited, together to STAT3 phosphorylation [32]. These data confirm some previous observations where RSV induced apoptosis of adult T cell leukemia cells by down-regulating the antiapoptotic protein surviving [33].

As mentioned in the introduction, RSV also exerts its effects on cell metabolism. Consistently, many authors using different experimental models have reported that Adenosine Monophosphate-activated Protein Kinase (AMPK) is a key target in mediating RSV activity on metabolism; for instance, RSV stimulates glucose transport in myotubes by activating AMPK [34].

An interesting *in vivo* study demonstrates that mice deficient for AMPK are insensitive to RSV-mediated metabolic effects [35]. Furthermore, the lifespan extension of worms mediated by RSV requires AMPK [36].

Recently, an intriguing relationship between RSV, adiponectin and AMPK activation has been demonstrated by Wang and co-workers in 3T3-L1 adipocytes [37]. Adiponectin is an adipocyte-derived hormone that plays a relevant role in regulation of insulin sensitivity and energy homeostasis. In this work the results confirm the RSV-mediated increase and multimerization of adiponectin and the RSV-mediated increase of DsbA-L (a main modulator of adiponectin) in 3T3-L1 adipocytes. Interestingly, the authors demonstrate that the positive effects of RSV are mediated through the activation of AMPK and the transcription factor FOXO1 also in this adipocyte setting.

A new and intriguing activity of RSV has been demonstrated in Chronic Myelogenous Leukemia (CML) cell lines [38,39]. CML is characterized by the reciprocal chromosomal translocation t(9;22) (q34;q11) that results in the formation of the Philadelphia (Ph) chromosome [40]. The Ph chromosome (present also in the Ph+ ALL) contains the abnormal fusion gene *Bcr-Abl* that produces the fusion protein BCR-ABL. This abnormal product constitutively localizes in the cytoplasm and retains the tyrosine-kinase activities of the c-ABL enzyme therefore activating a cascade of pathways promoting the cell proliferation and the anti-apoptotic mechanisms. Notably, two major survival and proliferation pathways are activated by BCR-ABL tyrosine-kinase: the PI3K/AKT/mTOR and the Mitogen Activated Protein Kinases (MAPK) pathways respectively [40].

RSV is able to inhibit the growth of CML leukemic cells by means of different mechanisms. One way is by activating AMPK that is a metabolic sensor at the crossroads between DNA damage and cell growth regulation [41]. AMPK is recognized as one of the main suppressors of the subunit mTORC1, a heterotrimeric protein kinase that includes mTOR [42]. AMPK is activated by RSV also in CML and participates in two relevant steps leading to the inhibition of the mTOR pro-survival pathway. First, AMPK activates tubular sclerosis 1–tubular sclerosis 2 (TSC1/2) heterodimer leading, in turn, to the inhibition of Ras homologue enriched in brain (Rheb) [41]. Rheb is a small GTP-binding protein that activates mTORC1. The second mechanism by means of which AMPK inhibits mTORC1 is through the RSV-mediated activation of autophagy in CML cells [39,43]. AMPK phosphorylation on Thr172 is increased following RSV treatment in both Imatinib-sensitive and Imatinib-resistant CML cell lines [39]. This is accompanied by the decrease of the phosphorylation status of mTOR, p70-S6 kinase, S6 ribosomal protein and 4-EBP1, suggesting the blockade at the level of TSC1/2, the heterodimer that inhibits mTORC1. The knockdown of AMPK in CML cells leads to the abrogation of the RSV-mediated LC3-II accumulation. LC3-II is a hallmark of autophagy that is up-regulated by RSV treatment. Coherently, the constitutive expression of mTOR upon engineering in the same cells abrogates the RSV-mediated LC3-II accumulation as well. These experiments show that RSV may regulate autophagy in CML cells through the activation of AMPK and the inhibition of the mTOR pathway [39].

Consistent with this evidence, a new population-based genetic association study has recently unveiled a role for the AMPK subunit haplotype in the risk to develop NHL in women with no family history of cancer [44]. Specifically, the association of two haplotypes with follicular lymphoma (FL) and diffuse large B-cell lymphoma (DLBCL) histological subtypes strengthens the link between AMPK and lymphoma pathogenesis also in humans.

It has also been reported that human B lymphoma cells treated with RSV up-regulate the class-II human leucocyte antigen (HLA-II) [45]. This phenomenon involves both classical and non-classical HLA class-II proteins and leads to the increase in the HLA class-II antigen processing in B-cell lymphomas and their subsequent recognition by CD4^+^ T cells. These data suggest that RSV may be useful in improving the immune recognition of malignant B cells by CD4^+^ T lymphocytes, opening an interesting perspective for the immunochemotherapy of B-cell lymphomas.

A recent work by Espinoza and co-workers describes a new property of RSV in leukemia cells [46]. The activating receptor NKG2D is expressed by cells of the innate and adaptive immune system, including the Natural Killer (NK) cells. NKG2D promotes the cytotoxic lysis of cancer cells by interacting with diverse and structurally different ligands. Several leukemia cell lines express the NKG2D ligand (NKG2D-L). This ligand, when up-regulated by stress stimuli, confers to the ligand-expressing cells a higher susceptibility to the NK-mediated cell lysis through the NKG2D receptor [47]. A major modulator of the expression of NKG2D-L at the cell surface is ataxia-telangiectasia mutated (ATM) [48]. The experiments performed by Luis Espinoza and colleagues demonstrate several effects depending on the treatment with RSV. First, ATM is activated by RSV in leukemia cells and different NKG2D-Ligands (NKG2D-Ls) are up-regulated as well at the cell surface; Second, ATM knockdown through shRNAs blocked the RSV-mediated up-regulation of surface NKG2D-Ls; Third, the NK-mediated cell death of leukemia cells is enhanced by pre-treatment with RSV and this effect is proportional to the levels of NKG2D-Ls induced by RSV in different leukemia cell lines [46]; Therefore, the NKG2D-triggered cell death may be a therapeutic mechanism elicited by the treatment with RSV on target cells also *in vivo*. The involvement of the immune system effector cells would be a great enhancement that cooperates and synergizes with the direct inhibitory action of RSV on cancer cells.

The chemopreventive properties of RSV have been studied also in terms of the potential to modulate estrogen homeostasis and, consequently, to inhibit the formation of estrogen-DNA adducts [49]. The formation of estrogen-DNA adducts is recognized to be a critical factor in the etiology of several human cancers, including NHL. Specifically, a study conducted on men diagnosed with NHL shows that the concentration of estrogen metabolites, conjugates and depurinating DNA adducts in the urine was several folds higher in NHL patients (*n* = 15) than in healthy control men (*n* = 30; median ratio of 86.0 *vs.* 18.0, respectively) [50]. The general mechanism of estrogen-DNA formation involves the oxidation of catechol estrogens to quinones, which can react with DNA. The excessive formation of catechol estrogen quinones can lead to cancer initiation. Balanced and unbalanced estrogen homeostasis can be preserved or mitigated, respectively, by the use of specific compounds, including RSV. RSV reduces the semiquinones to catechol estrogens and leads to the reduction of the amount of catechol estrogen quinones available to react with DNA in order to form the critical adducts for cancer initiation [49].

**Figure 1.**
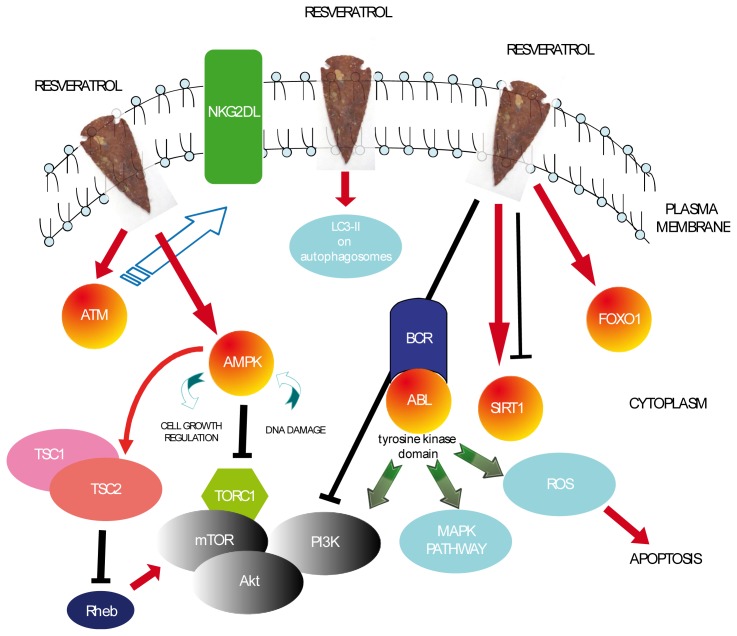
Schematic representation of the recently discovered RSV molecular targets on leukemia or lymphoma cells as described in this paper (ATM, Ataxia telangiectasia mutated; NKG2DL, killer cell lectin-like receptor subfamily K member 1-ligand; LC3-II, microtubule-associated protein 1 light chain 3; AMPK, Adenosine Monophosphate-activated Protein Kinase; TSC1/2, tubular sclerosis 1–tubular sclerosis 2; Rheb, Ras homologue enriched in brain; mTOR, mammalian target of rapamycin; TORC1/2, target of rapamycin complex 1/2; BCR-ABL, breakpoint cluster region protein-c-abl oncogene 1, non-receptor tyrosine kinase; SIRT1, silent information regulator 2 homolog 1; FOXO1, forkhead box protein O1; ROS, reactive oxygen species; MAPK, mitogen-activated protein kinase; PI3K, phosphatidil-inositol 3 kinase; Akt, RAC-alpha serine/threonine-protein kinase). The arrows represent an activation while the T-shaped lines represent an inhibition.

The main molecular targets described in this paragraph are represented in Figure 1. Collectively, the reported evidence supports further studies on human subjects and, specifically, on lymphoma and leukemia patients.

## 3. RSV in Hodgkin Lymphoma

There is currently just one paper describing the effects of RSV in Hodgkin lymphoma (HL) [51]. In this work we assessed the RSV potential to induce apoptosis and inhibit the cell cycle progression of the L-428 HL cell line. We demonstrated the dose-dependent, pro-apoptotic activity and the involvement of some molecular mediators such as SIRT1, p53 and FOXO3a as caused by RSV treatment. We also showed, for the first time, the anatomical localization of the histone/lysine deacetylase SIRT1 in human reactive lymph nodes and in HL-affected lymph nodes on a total of 30 patients. These preliminary observations suggest a selective expression of SIRT1 in the germinal centers (GCs) of the follicles and in the Hodgkin Reed-Sternberg cells, respectively. The GCs are the areas of the follicles where the lymphocytes proliferate after the antigen encounter. Together with the affinity maturation of antibodies, the GC reactions also bear the risk of generating autoreactive B-cells and malignant B-cell clones [52].

Furthermore, our preliminary observations point to the fact that SIRT1 is highly expressed by proliferating centroblasts (unpublished data). This can be due either to the fact that actively proliferating cells feature a higher metabolic rate when compared to resting cells, or to the direct involvement of SIRT1 during the proliferating process.

The fact that SIRT1 is activated or inhibited in cancer cells by RSV is still a matter of debate (Figure 1). SIRT1 can block senescence, cell differentiation and stress-induced apoptosis while promoting cell growth and angiogenesis [53]. Yet, there is also evidence pointing to the potential of SIRT1 to suppress the growth of intestinal and colon cancers, among others [54]. The current opinion is that the protein level and the enzymatic activity of this deacetylase can be modulated in a context-dependent fashion and that SIRT1 is involved in carcinogenesis, even though the mechanism still remains elusive [55].

## 4. *In Vivo* Tumor Experimental Models

RSV has been investigated in terms of therapeutic and chemopreventive potential in several *in vivo* cancer models. These experimental models encompass tumors of epithelial origin and just a few hematologic tumors. Some of the most relevant challenges on tumors of epithelial origin are summarized in Table 1 [17,56–65].

The aim of this paper is not a comprehensive summary of all the *in vivo* experimental cancer models where RSV has been tested. On the contrary, our focus is to underline the most relevant and up to date findings available that provide useful information in support of the use of RSV for the treatment of hematological tumors.

The literature reports the data on mouse lymphocytic leukemia both *in vitro* and *in vivo* [67]. This paper describes the antiproliferative and pro-apoptotic activity of RSV on mouse lymphocytic leukemia and translates the model system into BALB/c mice. The *in vivo* reported results show the increase in the overall survival of tumor-bearing mice after RSV intra-gastric administration (Kaplan-Meier curves). Spleen lymphocytes also showed a higher Con A-induced proliferation rate after treatment with RSV when compared to the controls. In this work, the effect of the intraperitoneal injection of RSV on the survival of the mice is not reported though, at variance with the results shown for the intra-gastric administration).

Mouse T-lymphoma EL-4 cells were injected into immunocompromised NOD/SCID mice and treated with RSV (100 mg/kg body weight) [68]. The results demonstrate the dose-dependent reduction of the tumor mass and the increase of the overall survival when the mice received daily doses of RSV orally. These interesting results are supported by the findings that RSV induced apoptosis of EL-4 cells via both the intrinsic and the extrinsic apoptotic pathways [68].

The *in vivo* evidence concerning Acute Lymphoblastic Leukemia (ALL), on the contrary, do not demonstrate any beneficial effect of the oral or intraperitoneal administration of RSV to immunocompromised NOD/SCID mice engrafted with this human leukemia [69,70]. When mice were fed with a RSV-containing diet and next challenged with ALL engrafment, no delay in leukemia development was observed. Furthermore, RSV did not improve the activity of the chemotherapeutic drug vincristine since the survival curves and the percentages of human lymphocytes in the blood showed no differences with the controls [69]. When given intraperitoneally, RSV did not have any positive effect on the progression of ALL neither in terms of survival curves nor percentage of circulating human leukemic cells [70].

Another mouse challenge of RSV on Balb/c mice injected with human acute myeloblastic leukemia Kasumi-1 cells reports a significant improvement of survival of the animals treated with RSV [71]. In this model, RSV was administered i.g. for 24 days before the injection of Kasumi-1 leukemia cells through the tail vein. The differences were statistically significant when the animals were administered with 10 or 20 mg/kg/day of RSV. Phospho-STAT3 was decreased by RSV in the livers of the treated animals when compared to the controls.

This data concerning lymphoma or leukemia experimental models (summarized in Table 2) strengthens the need to carefully choose the route of administration during the challenges and point to the fact that the outcomes may be context-dependent.

## 5. RSV and Challenges in Humans

The literature that concerns the effects of RSV on human subjects presents significant findings but is limited thus far [74].

Two of the most relevant challenges on cancer are represented by the works by Brown and Patel [75,76]. These studies described the administration of RSV orally to twenty human subjects with histologically confirmed colorectal cancer at eight daily doses before surgical resection. The administration of RSV caused the reduction of the tumor cell proliferation by 5% (*p* = 0.05) and this effect was likely due to the parent compound and by resveratrol-3-*O*-glucuronide that were recovered from the tissues [76]. Parallel research was aimed at evaluating the safety, the pharmacokinetic and the effects on circulating levels of insulin-like growth factor-1 (IGF-I) and IGF-binding protein-3 (IGFBP-3) after repeated dosing of RSV. This research describes the study on forty healthy volunteers ingesting RSV daily for 29 days. The results demonstrate that resveratrol-3-*O*-sulfate, resveratrol-4′-*O*-glucuronide, and resveratrol-3-*O*-glucuronide are major plasma metabolites and, most importantly, that the ingestion of RSV caused a decrease in circulating IGF-I and IGFBP-3 (*p* < 0.04 for both) in all volunteers. Therefore, repeated administration of high doses of RSV generates micromolar concentrations of parent and much higher levels of glucuronide and sulfate conjugates in the plasma, together with a decrease in circulating IGF-I and IGFBP-3. The modulation of these molecules in the plasma might contribute to cancer chemopreventive activity since the IGF signalling system (consisting of IGFs, IGFBPs and IGF receptors) plays a role in tumorigenesis [75]. Specifically, several studies suggest a direct relationship between levels of IGF-I, and an inverse relationship between the levels of IGFBP-3, and the risk of colorectal, prostate, breast or lung cancer [77]. Interestingly, the anticarcinogenic activity of calorie restriction in preclinical models seems to be, at least in part, mediated via the reduction of circulationg IGF-I [78]. It has been published that RSV can lower circulating IGF-I in diabetic mice on a high-calorie diet and in prostate tumor tissue of TRAMP mice [79,80]. These data collectively represent a link between RSV chemopreventive activity and IGF signalling system also in human cancer.

Very relevant information concerning RSV sulphate-metabolites is reported in a recent paper by Patel and colleagues [81]. Here, the authors demonstrate that RSV metabolites contribute to the *in vivo* activity through regeneration of the parent compound. After repeated ingestion of RSV by healthy volunteers and cancer patients, sulphate and glucuronide conjugates of RSV in human plasma and tissue were measured. The extent of the cellular uptake dictated the antiproliferative activity and relies on specific membrane transporters. Furthermore, colon cancer cell lines incubated with RSV-monosulfate mixture (75 μM) or with RSV (10 μM) generate RSV and resveratrol-3-*O*-sulfate, respectively, within the cells. Growth inhibition was observed after RSV-monosulfate mixture administration and, most interestingly, sulphate metabolites induced autophagy and senescence. Lipid-bound protein 1 light chain 3 (LC3-II, a marker of autophagy) and the cyclin-dependent kinase inhibitor p21 (a marker of senescence) decreased intracellularly after the treatment with a steroid sulfatase inhibitor (estrone 3-*O*-sulfamate), even after the treatment with RSV-monosulfate mixture. These data indicate that RSV regeneration within the cells is a pivotal event for the observed antiproliferative activity. Therefore, sulphate-conjugates represent a physiological circulating pool of RSV that gradually regenerates the parent compound in selected cells and tissues, leading to the *in vivo* observed beneficial effects [81].

Another paper reports the effects of RSV on human PBMCs isolated from healthy volunteers [72]. This interesting work demonstrates the bi-phasic effect of RSV on human B-cell proliferation. Specifically, PBMCs were analyzed after six days of culture and assessed for their proliferation potential. RSV doses of 5 μM increased the proliferation of CD19^+^ B lymphocytes in a statistically significant manner while a concentration of 10 μM inhibited B lymphocyte proliferation. These data suggest that RSV affects human B lymphocyte proliferation and apoptosis *in vivo*.

An intriguing antileukemic activity of RSV on human chronic lymphocytic leukemia (CLL) patients was recently been reported by Tomic and co-workers [73]. CLL patients were administered with RSV and some haematological and molecular parameters of circulating tumor cells were measured. STAT3 phosphorylation decreased, as well as white blood cells count and *O*-linked β-*N*-acetylglucosamine (*O*-GlcNAc) proteins. CLL cells are characterized by high levels of *O*-GlcNAc proteins and these affect intracellular signalling processes and disease progression [82]. This study is limited though by the very low number of CLL patients tested (*n* = 3) and by the poorly defined schedule of administration of RSV to the patients.

## 6. Conclusions and Perspectives

RSV is an attractive molecule in several fields of human health, as confirmed by the number of publications concerning this natural phytoalexin [74]. Cancer is, among the age-related deadly diseases, one of the research fields where RSV has demonstrated a great potential but has been just partially characterized, possibly due to the low bioavailability of the parental compound together with the partial knowledge of the role played by RSV-glucuronides and -sulfonates that are generated physiologically in the bloodstream [81,83].

The low bioavailability of the parental compound based on poor resorption and extensive biotransformation is a well known limit of the molecule [84]. The metabolic pattern of RSV is complex and leads to the formation of 3-sulfate, 3,4′-disulfate, 3,5-disulfate, 3-glucuronide, 4′-glucuronide and two diglucuronides, as described by Burkon and Somoza [85]. It seems also that, in the intestine and liver, the dose of RSV dictates the type of produced metabolites. Specifically, at low RSV concentrations, sulfation prevails whereas at higher RSV concentrations sulfates drop and glucuronides become prevalent [86,87]. RSV exerts several *in vivo* activities despite the extensive biotransformation and this may be explained through some mechanisms including the hydrolysis of the conjugates in the target tissues (that re-generates the parent compound), the recirculation after deconjugation in the gut and the biologic activity of the RSV-sulfates and RSV-glucuronides themselves [84].

Another interesting RSV-related molecule is piceatannol, which is a naturally occurring stilbene present in sugar cane, berries, peanuts, red wines and the skin of grapes [88]. Piceatannol is also a RSV-metabolite, generated via the cytochrome P450 1A2 and 1B1 enzymes and it is one of the main RSV-metabolites present in the liver. Therefore, one hypothesis is that RSV may function as a pro-drug for the production of piceatannol or other stilbenes that probably contribute to the observed beneficial effects [88].

The clinical trials in human subjects focused on diabetes, cardiovascular diseases, cancer and inflammation and the doses of RSV administered were diverse. The described ranges span from 0.03 to 5.0 g daily doses of RSV [74]. RSV is rapidly absorbed when administered orally and reaches a peak in the bloodstream within 60 min [75,76]. However, the already mentioned rapid conversion to different metabolites hampers the definition of a suitable, recommended dose for a given disease. The conclusions reached by the working group on RSV on this issue are that the relevant effective doses of RSV need to be established in humans in relation to the different diseases that it may counteract and that these doses will vary accordingly to the specific effects being studied [74].

The confirmed sensitivity of lymphoma and leukemia cells to RSV, together to the better knowledge of the actual molecular targets of this polyphenol, may lead to future useful applications. It is known that RSV:

- may exert its activity on human PBMCs in a bi-phasic/dose-dependent way;
- is cytotoxic to lymphoma and leukemia cancer cells since it can trigger apoptosis, autophagy or senescence;
- seems not to be toxic to human PBMCs, either resting or mitogenically stimulated;
- is well tolerated by humans and its physiological metabolites may work as a circulating reservoir of the parent compound that can be re-generated within the cells of the intestinal tract;
- is a chemically well-known molecule whose structure can be improved and modified by substitutions aimed at increasing the anticancer properties or the bioavailability of the active principle.

The recent findings reported in this paper provide a rational summary of some the most interesting molecular targets of RSV in lymphoma and leukemia. These should be integrated to the information provided by the most advanced techniques of Gene Expression Profiling and Next Generation Sequencing.

Indeed, the effects of RSV on human cells must be considered context-dependent and normal cells could respond differentially when compared to cancer cells to the same RSV doses and in the same experimental conditions. Even though this issue may increase the complexity, we believe that is a key feature of RSV that needs to be taken in consideration all the times this molecule is introduced in the experimental design.

Finally, more *in vivo* approaches are strongly recommended in order to define the actual chemopreventive or therapeutic doses that can be relevant and realistic for future developments. The optimal RSV dose that can achieve useful results has to be determined for each pathology and type of cancer and will possibly vary depending on the histology of the tumor. The same approach should be adopted during the characterization of RSV-derivatives as well as RSV-metabolites.

## Figures and Tables

**Table 1.** Some of the most relevant papers describing the use of RSV on animal experimental models of solid tumors.

Year	Tumor experimental model	Animal model	Reference
1997	Skin cancer	Female CD-1 mice	[56]
2009	Skin cancer	Female C3H/HeN mice	[57]
2009	Skin cancer	Female Swiss mice	[58]
2010	Skin cancer	SENCAR mice	[59]
2002	Breast cancer	Female Sprague-Dawley rats	[60]
2005	Breast cancer	FVB/N female mice	[61]
2013	Breast cancer	Female Sprague-Dawley rats	[62]
2013	Breast cancer	Female BALB/c mice	[63]
2006	Colorectal cancer	Male Wistar rats	[17]
2010	Colorectal cancer	C57 BL/6 mice	[66]
2009	Prostate cancer	Male Sprague-Dawley rats (SV-40 Tag)	[64]
2013	Lung cancer	Female SCID mice	[65]

**Table 2.** Some of the most relevant papers concerning *in vivo* challenges of hematological tumors with RSV.

Year	Tumor experimental model	Animal model	Reference
2007	L1210 mouse lymphocytic leukemia	Male BALB/c mice	[67]
2011	EL-4 mouse lypmhoma	NOD/SCID mice	[68]
2012	Human ALL	NOD/SCID mice	[69]
2012	Human ALL	NOD/SCID mice	[70]
2010	Human acute myeloblastic leukemia Kasumi-1	Male BALB/c mice	[71]
2009	Human PBMCs		[72]
2013	Human CLL		[73]
